# Neurosurgical Treatment of Breast Cancer Metastases to the Neurocranium

**DOI:** 10.4061/2011/549847

**Published:** 2010-12-16

**Authors:** Andreas M. Stark

**Affiliations:** Department of Neurosurgery, Universitätsklinikum Schleswig-Holstein, Campus Kiel, Arnold-Heller-Stra*β*e 3, 24105 Kiel, Germany

## Abstract

Breast cancer metastases to the neurocranium might involve the bone, the dura, or the brain parenchyma. The latter location is the far most common. The annual incidence of brain metastases in patients with breast cancer is in the range of 4–11 per 100.000 persons per year. Symptoms and findings mainly result from the location of the lesion. The diagnostic method of choice is magnetic resonance imaging before and after administration of contrast material. Breast cancer brain metastases present as solid, cystic, or partially cystic lesions with marked contrast enhancement and perilesional edema. The therapeutic option of choice is microsurgical resection whenever possible. Adjuvant treatment includes radiotherapy, radiosurgery, and/or chemotherapy.

## 1. Introduction

Breast cancer metastases to the neurocranium may involve the bone, the dura, or the brain parenchyma. The latter is the most common location. Affected patients are often in an advanced stage of disease [1]. According to advances in treatment of primary tumors, the amount of patients eligible for surgery is rising. 

As early as in 1889, Stephen Paget showed that “the distribution of the secondary growths is not a matter of chance.” Based on autopsy findings in 650 patients with breast cancer, he described affection of the cranium in 36 cases, whereas there was no single case of metastasis to the hand or feet [2]. It is an important clinical observation that tumors exhibit a predilection to metastasize to certain organs. Current research has addressed the molecular process of the metastatic cascade: (a) migration from the primary tumor, (b) dissemination into and survival in the blood vessels, (c) extravasation, and (d) proliferation at a distant site as well as the importance of the tumor-host interface [3–5]. 

In the first section of this paper, the general terms of neurosurgical treatment of metastases to the neurocranium from solid tumors are presented. In the second section, special issues of brain, bone, and dural metastases from breast cancer are discussed.

## 2. General Terms of Neurosurgical Treatment of Metastases to the Neurocranium

### 2.1. Clinical Examination

Diagnostic workup includes a complete neurological examination and evaluation of the extent of the primary tumor as well as comorbidity. Bone metastases might be detected by bulking of the calvaria. Brain metastases often lead to hemiparesis, ataxia, and aphasia [6]. Neurological examination can also give clues for possible spinal involvement. These patients might show ataxia and paresis and complain about radicular pain or sensation deficits.

### 2.2. Imaging

Magnetic resonance imaging (MRI) is useful for detecting small intraparenchymal lesions down to 1 mm diameter. Concerning this issue, MRI is highly superior to computed tomography (CT). Furthermore, MRI can sufficiently detect leptomeningeal spread and small dural lesions. It is very useful in detecting dural involvement in patients with bone or brain metastases. This information is important for surgery. MRI is usually generated in a sagittal, coronal, and axial view. 

CT is useful in determining the extent of bone destruction, either in bone metastases or in bone involvement resulting from metastases to the dura or brain.

Scintigraphy is used for screening purposes. If metastasis to the skull is suspected, CT scan should follow. If metastasis to the brain is suspected, MRI should follow.

**Figure 1:**
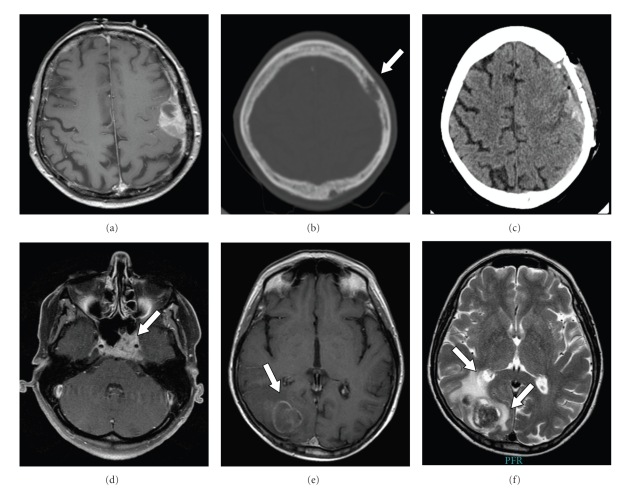
(a)–(c) 77-year-old femal with aphasia resulting from breast cancer dural metastasis, (a) preoperative T1-weighted MRI showing contrast enhancement of a left parietal dural tumor. (b) Preoperative CT scan shows bone erosion (arrow). (c) Postoperative CT scan documents the removal of the dural mass and the infiltrated bone which has been substituted by bone cement cranioplasty. (d) T1-weighted MRI showing contrast-enhancing breast cancer skull base metastasis in the clival region occurring in a 45-year-old female with known breast cancer and diplopia (arrow). (e, f) MRI of a 69-year-old female with right occipital breast cancer brain metastases. (e) T1-weighted MRI shows a ring-like contrast-enhancing lesion (arrow). (f) T2-weighted MRI shows the lesion with significant peritumoral finger-like edema (arrows).

Figure 1 shows examples for imaging results in patients with breast cancer metastases to the neurocranium.

### 2.3. Neuronavigation

Nowadays, preparation for surgery includes neuronavigation for most cases of intracranial tumors. Neuronavigation is a 3D computer model of the patient's head which can be used intraoperatively as a reference location system. Basically, a thin-slice MRI or CT scan is performed after reference markers have been applied to the patient's head (*fiducial markers*). The 2D data set is transferred to a working station and processed into a 3D set. Immediately before starting the operation, the position of the patient's head is registered to the system in relation to a reference star. Thus, the surgeon can control the location of a pointer tip intraoperatively. Neuronavigation is helpful in minimizing the approach to the tumor. In glioma surgery, it is also useful for resection control. It must be noticed that intracranial structures are “shifting” after opening of the skull and again after resection of intracranial lesions. This incident is called *brain shift*.

## 3. Breast Cancer Metastases to the Neurocranium

### 3.1. Skull Metastases

The rate of hematogenous skull metastases, even though it is low in comparison to brain metastases, is higher in breast cancer than in many other tumors [7]. Hopkins et al. have described that bone metastases were detected by 99 mTc scintigraphy in approximately 50% of breast cancer patients in an early stage of the disease [8]. However, most of the lesions do not become symptomatic. It can be estimated that they grow slowly allowing other complications of the underlying breast cancer to develop. Most patients present with local swelling, sometimes accompanied by local pain. Neurological deficits are infrequent at the time of presentation. Bone metastases may reach significant size until they become symptomatic and, thus, until they are diagnosed. Metastases of the calvaria are often noted by the patient or by the patients family as swelling [9, 10]. Skull base metastases may become symptomatic by diplopia and/or exophthalmia when the orbit is involved. CT is required for visualization of the extent of bone destruction. MRI is superior to CT in detecting infiltration of the dura and neural tissue. Thus, both techniques are required preoperatively. Surgery should be considered when (1) a neurological deficit is present and/or (2) massive destruction of bone (and dura) occurs, (3) when there is a painful mass, (4) when solitary metastasis is present, or (5) when confirmation of the diagnosis is warranted [10]. Surgery aims to resect the infiltrated bone and replace it by bone-cement (cranioplasty). Alternatively, a titan mesh may replace the bony defect. This is especially useful in complex lesions involving the skull base. If the dura is infiltrated, it needs to be both resected and replaced The differential diagnosis of skull metastases includes primary skull tumors (e.g., osteoma, chondrosarcoma, chordoma, dermoid, and epidermoid cysts) and benign tumor-like lesions (e.g., fibrous dysplasia, hyperostosis, and eosinophilic granuloma) [10].

### 3.2. Dural Metastases

Dural metastases are uncommon. According to a postmortem series of 27 patients, breast cancer was the second most common malignancy to cause dural metastases. The most frequent cancer type was prostate cancer. The third most common type was cervical cancer [11]. Dural metastases may become symptomatic as solid masses or as metastatic subdural fluid collection, assembling a chronic subdural hematoma. Dural metastases are an important differential diagnosis of meningioma and must be suspected in every patient with chronic subdural hematoma and underlying malignant disease [12–14]. When solid dural metastases are removed, the dura is incised circumferentially around the lesion. Then, the tumor is dissected from the brain tissue with cottonoids and gentle coagulation. Retraction of the brain should be avoided. Accompanying veins should be preserved. After circumferential dissection, the tumor can be gently removed. The dura has to be replaced by autologous graft tissue or artificial material. The intraoperative aspect of dural metastases is quite similar to that of meningioma. Histological examination is essential to clarify the diagnosis.

### 3.3. Brain Metastases

#### 3.3.1. Epidemiology

Breast cancer is the second most common solid tumor that forms brain metastases. The most common tumor type is lung cancer. The annual incidence of breast cancer brain metastases in USA is in the range of 5–10 per 100.000 per year. Altogether, 10–15% of patients with metastatic breast cancer will develop brain metastases during the course of the disease. The median age at time of diagnosis of breast cancer is 47 years. The mean latency between diagnosis of breast cancer and the detection of breast cancer brain metastases is 2-3 years. However, brain metastases may occur even as long as 20 years after diagnosis of breast cancer [1, 6, 15].

#### 3.3.2. Location and Symptoms

Brain metastases characteristically grow at the white/gray matter border. They are more frequently located in the supratentorial than in the infratentorial space. Symptoms depend on the size and the exact location of the lesion. Even small lesions may cause neurological deficits if they grow inside or close to eloquent brain areas as the motor area or the speech regions. Large lesions may cause mass effect by increasing the intracranial pressure or by blocking cerebrospinal fluid (CSF) pathways causing hydrocephalus. According to our own data including 47 patients, the most frequent symptoms at the time of presentation to a neurosurgical unit were ataxia (23%), headache (21%), visual disturbance (15%), hemiparesis (11%), and vertigo (11%) [6, 16]. In few cases, brain metastases are incidental findings during staging procedures.

In 2005, we published a series of 177 consecutive patients who underwent craniotomy for newly diagnosed brain metastases from various tumors. Following lung cancer, breast cancer was the second most common origin of brain lesions. Colorectal cancer and renal cancer were the third and fourth most common origins. The amount of solitary brain lesions (only detectable distant metastases in the body) was 26% in breast cancer lesions whereas it was 72% in nonsmall cell lung cancer (NSCLC), 29% in colorectal cancer (CRC), and 56% in renal cancer (RC). Extracranial metastases were present in 54% of breast cancer patients versus 45% in NSCLC, 59% in CRC, and 33% in RC. Synchronous diagnosis of the primary tumor and brain metastasis is very uncommon in breast cancer (3%, 1 patient). In contrast, it is common in NSCLC (54%) and CRC (59%). It is uncommon in renal cancer (11%) [6].

#### 3.3.3. Prognostic Indicators

Overall, accepted indicators of prolonged survival are only younger age and good patient performance. There is no uniform definition of elderly patients with brain metastases. Usually, the threshold between younger and elderly individuals is set somewhere between 50 and 75 years [17–19]. In a systematic statistical evaluation, we could define the age threshold relevant for prognostic differences as 65 years [16]. Patient performance is usually documented as Karnofsky performance score (KPS) as described by Karnofsky and Burchenal, in 1949 [18, 19]. Usually, a KPS of 70 or higher is regarded as favorable and is often required for the inclusion of patients into treatment studies. 

**Figure 2:**
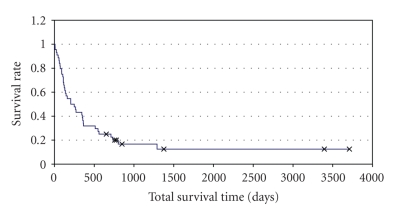
Kaplan Meier plots depict survival of 47 patients with breast cancer brain metastases who underwent surgical resection and adjuvant radiotherapy in our department between 1994 and 2004. 42 patients had one brain metastases, 4 patients had 2 intraparenchymal lesions, and 1 patient had 3 metastases. Median survival was 205 days = 29 weeks.

For clinical decision making, additional factors are taken into account. Herein, local control of the primary tumor is an important factor as well as the extent of extracranial metastases and the number of intracranial metastases [20–22].  Figure 2 shows the survival curve of 47 patients who underwent surgery for breast cancer brain metastases in our department in a 10-year interval.

#### 3.3.4. Imaging

The standard imaging technique for the detection of brain metastases is MRI before and after the administration of contrast material. Herein, brain metastases appear as ring-like, solid or partially solid/cystic contrast-enhancing lesions in T1-weighted imaging. The surrounding edema, which is often significant, can be best visualized in the T2-weighted image. It is a finger-like edema of the white matter. MRI is highly superior to (CT) in detecting small lesions down to 1 mm diameter. CT is adequate for the emergency situation and should be followed by MRI once brain metastases are suspected. Infrequently, patients with brain metastases present with acute, stroke-like symptoms caused by intratumoral bleeding. This condition is relatively frequent in renal cancer and malignant melanoma but may also occur in individuals with breast cancer.

#### 3.3.5. Surgery: Indications and Techniques

The goal of surgery in patients with brain metastases is to establish the diagnosis and relieve mass effect. If brain metastases are accessible, they should be completely removed. Currently, there is evidence that brain metastases, like gliomas, create an infiltration zone involving the adjacent brain parenchyma. It will be up to further studies to generate treatment decisions from this observation. 

Randomized prospective data concerning the surgical treatment of patients suffering from brain metastases are only available for patients with single brain lesions. Herein, it has been proven that surgery plus radiotherapy (external beam radiation) is superior to radiotherapy alone in prolonging patient's survival [23, 24]. 

Sufficient data for patients with multiple metastases are lacking. From the existing literature, it can be assumed that in patients with 1–3 brain lesions open microsurgical resection should be considered whenever possible. In this situation, surgical intervention is indicated in patients who present with (1) a primary tumor under control, (2) accessible brain lesions, and (3) a total number of brain metastases of at least 3 [18, 19]. As a rule of thumb, patients undergoing surgical resection of brain metastases should have a life expectancy of at least 6 months. However, occasionally, patients with multiple brain metastases present with one life-threatening lesion. This lesion might be resected in an emergency operation in order to prevent sudden patient death. In general, due to advances in operative techniques and neuroanesthesia, the removal of 1–3 brain metastases in 1-2 operations is appropriate. Small lesions (≤3 cm diameter, ≤3 metastases in total) might be treated by stereotactic radiosurgery as an alternative to open surgery or as additional treatment [6, 18, 19, 25]. 

In patients with multiple metastases, it has been shown that radiotherapy plus radiosurgery is superior to radiosurgery alone. As a consequence, radiosurgery should not be performed as single therapeutic method [26]. 

Preparation for surgery includes the selection of the approach which is in most times neuronavigation. Corticosteroids should be given to prevent or reduce brain swelling. The patient's head is placed in a rigid fixation system to prevent unwanted movement during the operation. Neuronavigation-guided skin incision and bone trepanation is performed. In superficial metastases, the dura should be inspected for possible infiltration. Infiltrated dura needs to be replaced. The intracranial part of the operation is carried out using the operative microscope. After dural opening, the metastasis is accessed and dissected from the surrounding brain tissue. In larger lesions, it may be necessary to partly remove the lesion before dissecting it. Frozen sections may be performed for intraoperative histology. Completely removing the metastasis without damaging the adjacent brain tissue should always be the goal. After removal of the lesion, meticulous hemostasis is performed by cauterization followed by the application of cellulose strips. After irrigation with saline, the dura is closed and the bone flap is placed and fixed. Tissue specimens are sent to histopathology for definitive diagnosis.

#### 3.3.6. Complications of Surgery

Brain metastases tend to cause brain swelling which can be treated by steroids and osmotic diuresis. Steroids should be continued over a few days after surgery and should then be reduced over some days before they are tapered. Prophylactic antibiotics can reduce infection. Careful microsurgical dissection prevents injury to the brain tissue and to small veins thus preventing unfavorable neurological outcome. Specific complications of surgery include intracranial bleeding (intraparenchymal, epidural, subdural), subtotal resection (if it is not primarily wanted), injury to eloquent brain areas and cerebrospinal fluid fistula. In patients who have had seizures prior to surgery, anticonvulsants should be given. Postoperatively, special care should be taken to prevent deep vein thrombosis.

#### 3.3.7. Complications of Brain Metastases: The Course of the Disease

As many as 50% of patients will suffer from brain metastasis recurrence—either at the site of the treated metastasis or anywhere else within the brain. Reoperation is possible. However, most patients fail to qualify for repeated surgery due to poor performance. 

Patients with breast cancer brain metastases tend to develop leptomeningeal dissemination (leptomeningeal carcinomatosis) more often than patients with brain metastases of other origin. Leptomeningeal spreading is caused by dissemination of tumor cells via the cerebrospinal fluid (CSF). It is best visualized with contrast-enhanced MRI. Treatment might consist in intrathecal chemotherapy given via an implanted reservoir. Herein, methotrexate is commonly used [27]. Radiotherapy might be an alternative or additional palliative option [28].

#### 3.3.8. Adjuvant Treatment

Radiotherapy is applied on a routine basis after surgical resection. Radiosurgery is an option for small lesions (≤3 cm diameter) either as an alternative to open resection or as additional treatment.

Chemotherapy for brain metastases is mostly targeted at the primary tumor. Delivering systemic chemotherapy to brain metastases is highly limited by the presence of the blood-brain barrier. Recent evidence highlights the importance of the alkylating agent temozolomide in patients with newly diagnosed brain metastases from breast cancer [29]. Temozolomide is applied orally and is well tolerated even by elderly patients. It is widely applied in the treatment of malignant gliomas.

#### 3.3.9. Postoperative Followup

Patients with brain metastases should be followed closely by a neuro-oncology/neurosurgery unit. Following surgery and radiotherapy, we look after patients in our outpatient department every three months. Herein, clinical examination and cranial MRI are performed. If brain metastasis recurrence is noted, recraniotomy is discussed. Further options include radiosurgery and chemotherapy depending on the primary tumor.

## 4. Conclusions

Breast cancer metastases to the neurocranium might involve the bone (either the calvaria or the skull base), the dura (either as a solid mass or as subdural fluid collection), or the brain parenchyma. The latter location is the far most common. The diagnostic method of choice is magnetic resonance imaging. The treatment consists in neuronavigation-guided microsurgical removal whenever possible. The decision whether to operate depends mainly on the stage of the disease, the number of brain lesions, and the performance status of the patient. Postoperative treatment consists in radiotherapy and/or chemotherapy depending on the primary tumor. Radiosurgery may be an alternative to surgery or can be added to surgical treatment in selected cases.
